# Changes in Rumen Bacterial Community Induced by the Dietary Physically Effective Neutral Detergent Fiber Levels in Goat Diets

**DOI:** 10.3389/fmicb.2022.820509

**Published:** 2022-04-11

**Authors:** Benchu Xue, Mei Wu, Shuangming Yue, Anhai Hu, Xiang Li, Qionghua Hong, Zhisheng Wang, Lizhi Wang, Quanhui Peng, Bai Xue

**Affiliations:** ^1^Animal Nutrition Institute, Sichuan Agricultural University, Chengdu, China; ^2^Department of Bioengineering, Sichuan Water Conservancy College, Chengdu, China; ^3^Yunnan Academy of Animal Science and Veterinary Medicine, Kunming, China

**Keywords:** goat, particle size, peNDF, rumen bacteria, growth performance

## Abstract

Physically effective neutral detergent fiber (peNDF) is a concept that accounts for the particle length of NDF in a feed, sustaining the normal chewing behavior and rumen fermentation of ruminants. This study aimed to elucidate the effects of dietary peNDF on growth performance and bacterial communities in the rumen of goats through a high-throughput sequencing technique. A total of 30 male Lezhi black goats were randomly assigned to five groups, corresponding to five diets with identical compositions and nutrient levels but with varying forage lengths (the peNDF_1_._18_ contents of the diets were 33.0, 29.9, 28.1, 26.5, and 24.8%, respectively). The whole trial lasted for 44 days. As results show, feed intake and average daily gain were highest when peNDF_1_._18_ content was 26.5%, in which the papilla length of the dorsal sac in rumen was the highest. Chao1 and ACE indexes were similar among the treatments, while Shannon and Simpson indexes of the peNDF_1_._18_ = 28.1% group were the highest (*p* < 0.05). As the level of dietary peNDF_1_._18_ decreased, the dominant phylum transitioned from Bacteroidetes to Firmicutes. The top three dominant genera of rumen bacteria were *Prevotella 1*, *Ruminococcaceae NK4A214 group*, and *Christensenellaceae R-7 group*. They all showed a quadratic correlation with dietary peNDF_1_._18_ level (*p* < 0.05). The relative abundance of *Ruminococcaceae UCG-011* was positively correlated, while that of *Prevotella 1* was negatively correlated, with amino acid metabolism and energy metabolism (*p* < 0.01). In conclusion, dietary peNDF level influenced goat growth performance, rumen development, and rumen bacterial community structures, and a peNDF1.18 level between 26.5 and 28.1% was considered optimal for goat diet.

## Introduction

Physically effective neutral detergent fiber (peNDF) is a concept proposed by Mertens (1997), which reflects the particle size (PS) of feed and the impact of feed on chewing activities and the ability to stimulate rumen stratification (Zhao et al., 2010). Ruminants need a suitable level and length of fiber to maintain the normal rumen pH. Effects of peNDF on the growth performance and nutrient digestibility of goats have been assessed in a previous study (Jang et al., 2017). Park et al. (2014) found that feeding growing steers with a low-peNDF diet could increase feed intake, thereby increasing daily gain. Likewise, Cao et al. (2021) found that nutrient digestibility and milk production dramatically improved with increased peNDF content. Through *in situ* assessment, Yang and Beauchemin (2007) found that the ruminal degradation rates of dry matter (DM), organic matter (OM), NDF, acid detergent fiber (ADF), starch, and nitrogen in the high-peNDF group were higher than those in the low-peNDF group. It was documented that reducing the PS of diet expanded the contact area between microorganisms and feed (Zhao et al., 2010) and therefore increased nutrient digestibility and reduced chewing time in dairy goats (Li et al., 2014). Beauchemin and Yang (2005) found that the long fiber particles (> 1 cm) promoted chewing and saliva secretion of dairy cows, which were essential for maintaining the stability of the rumen environment. Zebeli et al. (2012) proposed that the suitable level of peNDF_1_._18_ in the diet of dairy cattle should be around 31.2%. However, Zhao et al. (2010) found that 22.4% peNDF_1_._18_ was more conducive to the nutrient digestion of goats than was 43.1%. The difference in the optimal dietary peNDF level between cattle and goats suggested the inconsistent requirements for dietary peNDF level.

Ruminal microorganisms help their host in degrading fiber more successfully than do monogastric animals, which is of great value (Li et al., 2018b). Gastrointestinal microbes are affected by many factors, including diet, host genetics, living environments, and age (Jin et al., 2016). Studies have proved that changes in diet affect the gastrointestinal microbes in cattle (Klieve et al., 2007) and dairy cows (Valizadeh et al., 2010). However, few reports concerning the impact of peNDF on the rumen bacterial community in goats have ever been reported. Previous studies revealed that the counts of total bacteria (Valizadeh et al., 2010) and *Ruminococcus albus* (Zebeli et al., 2008) were not affected by dietary PS. Huo et al. (2014) discovered that a high-grain diet influenced the liquid- and solid-associated rumen bacteria of goats, but a high-forage diet increased the relative abundance of *Prevotella*.

Until now, there has been no research concerning the effects of the PS of fiber in diets on solid-associated bacteria in the rumen of goats. We assumed that changes in dietary peNDF levels could affect growth performance, rumen development, and rumen microbial structure in goats with the same dietary compositions and nutrient contents. The main objective of this study was to evaluate the effects of dietary peNDF level on the diversity and composition of rumen bacteria, which could explain the differences in growth performance in goats, based on which a reasonable range of dietary peNDF was proposed.

## Materials and Methods

### Ethics Statement

The experiment was approved by the Animal Policy and Welfare Committee of Animal Nutrition Institute, Sichuan Agriculture University, and followed the current laws of animal protection (Ethics Approval Code SCAUAC201408-3).

### Animals and Diets

The study was carried out at the Experimental Base of Animal Nutrition Institute (30.3°N, 103.0°E), Sichuan Agricultural University. Thirty male 4-month-old Lezhi black goats (purchased from the farm of Tianlong Agriculture and Animal Husbandry Technology Co. Ltd.) with similar body weights (BWs; 21.4 ± 0.24 kg) were randomly divided into five groups corresponding to five dietary treatments. The diets (Supplementary Table 1) were formulated according to the nutrient requirements of a 20-kg goat with a 150-g daily gain based on National Research Council (NRC) (2007) and were identical in composition and nutrient content but varied in length of roughage. The forages (alfalfa hay, peanut vine, and *Leymus chinensis*) were cut into 1, 5, 1, 4, and 7 cm, respectively, by a forage cutter-FS60, purchased from Nongfengli Machinery Equipment Co., Ltd (Jining, China). Forage PS distribution was determined using the Penn State Particle Separator, as reported by Kononoff et al. (2003). Pef_8.0_ and pef_1.18_ were calculated as the proportions of the DM of forage particles retained on 19-mm and 8-mm sieves and those on 19-mm, 8-mm, and 1.18-mm sieves of total DM content, respectively. The measured peNDF_1_._18_ contents of the diets were 24.8% (1 mm, L), 26.5% (5 mm, ML), 28.1% (1 cm, M), 29.9% (4 cm, MH), and 33.0% (7 cm, H), respectively (Table 1).

**TABLE 1:** PS distribution and peNDF content of five groups.

Items	Content of peNDF^a^				
	H (33.0%)	MH (29.9%)	M (28.1%)	ML (26.5%)	L (24.8%)
**% DM retained on sieves**					
19 mm	27.3	11.2	7.0	0.0	0.0
8 mm	9.7	11.8	9.3	6.3	3.1
1.18 mm	39.9	46.8	49.4	55.5	54.6
Pan	23.1	30.2	34.4	38.3	42.3
**Physically effective factor^b^**					
pef_8.0_	37.0	23.0	16.3	6.3	3.1
pef_1.18_	76.9	69.8	65.6	61.7	57.7
**peNDF content (%, DM)^c^**					
peNDF_8_._0_	15.9	9.8	7.0	2.7	1.3
peNDF_1_._18_	33.0	29.9	28.1	26.5	24.8

*^a^The different peNDF_1_._18_ (PS > 1.18 mm) contents of 33.0, 29.9, 28.1, 26.5, and 24.8% were obtained by crushing the forage into 7 cm (H), 4 cm (MH), 1 cm (M), 5 mm (ML), or 1 mm (L).*

*^b^Physical effectiveness factor determined as the proportion of the DM of particles retained on two sieves (19 mm and 8 mm) or on three sieves (19 mm, 8 mm, and 1.18 mm), respectively.*

^c^peNDF content, physically effective factor * NDF; peNDF_8_._0_ and peNDF_1_._18_, physically effective NDF determined as NDF content of ration sample multiplied by pef_8.0_ and pef_1.18_, respectively.

During the whole period, each goat was reared in a single metabolic cage in the same barn. The concentrate and the forage were evenly mixed and then fed to goats at 08:00 and 20:00 at equal amounts, allowing about 10% orts. Goats were free to access fodder and water. The leftovers were weighed at 8:00 every morning to calculate dry matter intake (DMI). The experiment lasted for 44 days, including a 14-day adaptation period and a 30-day experimental period. The BW of each goat was recorded on the 1st and 30th days of the formal trial using an electronic scale. Average daily gain (ADG) was calculated as ADG = (Final BW – Initial BW)/30 (kg/day).

### Sample Collection

At the end of the formal trial and before morning feeding, 15 goats were selected for slaughter after electric shocks. For each sampling, 300 g of whole rumen contents was obtained. A portion (∼50 g) of the whole rumen sample was homogenized on ice for three 1-min cycles at 1-min intervals using a Polytron grinding mill (Thermo Fischer Scientific, France). Approximately 0.5 g was transferred into 2-ml Eppendorf tubes and stored at −80°C until molecular biology analyses (Silberberg et al., 2013). After the experiment, the rest of the goats continued to be cultivated normally. The dorsal sac and ventral sac of rumen were collected from the same site and fixed in 4% paraformaldehyde for determination of tissue morphology.

### Body Weight and Average Daily Gain

The BW of each goat was recorded on the 1st and 30th days of the formal trial using an electronic scale. ADG was calculated as ADG = (Final BW - Initial BW)/30 (kg/day).

### Determination of Morphology of Rumen Epithelium

The rumen epithelial tissue was sent to Wuhan Service Biotechnology Co., Ltd. (Wuhan, China) for H&E staining and sectioning. Image-Pro Plus 6.0 was used to verify the qualified sample, with millimeters as the standard unit. The thickness of the five muscle layers, the width of the nipple, and the height of the nipple in the rumen epithelial section were measured at a 20-fold scale. Photographs were recorded at the same time.

### DNA Extraction and PCR Amplification and Sequencing

The DNA extraction kit (Tiangen Biochemical Technology, Peking, China) was used to extract the total DNA from the rumen chyme samples of goats, as described previously (Wang et al., 2019). The purity and concentration of the extracted DNA were detected by agarose gel electrophoresis. With the extracted DNA (1 ng/μl) as template, the V4–V5 region of the 16S rRNA gene was amplified by PCR with bacterial universal primers 515F (5′-GTGYCAGCMGCCGCGGTAA-3′) and 926R (5′-CCGYCAATTYMTTTRAGTTT-3′) (Parada et al., 2016). The PCR system (25 μl) consisted of 1 ng/μl DNA template (10 μl), 1 × PCR buffer (2.5 μl), 1.5 mM MgCl_2_ (1.5 μl), 0.4 μM dNTPs (2.5 μl), 1 μM upstream and downstream primers (1.5 μl each), 0.5 U of KOD-Plus-Neo enzyme (TOYOBO) (0.5 μl), and water (5 μl, added up to 25 μl). PCR procedure was as follows: predenaturation at 94°C for 1 min for 30 cycles (denaturation at 94°C for 20 s, annealing at 54°C for 30 s, and extension at 72°C for 30 s) and extension at 72°C for 5 min. PCR products were electrophoresed using a 2% agarose gel, recovered (using a gel recovery kit, Qiagen), and purified. 16S rRNA high-throughput sequencing (Rhonin Biotechnology Ltd., Chengdu, China) was performed after the tests were qualified by the Hiseq 2500 PE 250 sequencing platform.

### Sequencing Data Analyses

The sample data were distinguished according to the Barcode sequence, and the chimera was filtered by the Uchime algorithm to obtain clean data (effective data) (Knight, 2011). Uparse (V7.0.1001)^1^ was used to cluster all samples with 97% identity for operational taxonomic units (OTUs) (Edgar, 2013). The sequence with the highest frequency in OTUs was selected as the representative sequence of OTUs. Using UCLUST (DiTullio et al., 2000) to process the representative sequences of OTUs and comparing them with the SILVA132^2^ database (Quast et al., 2012), the taxonomy annotation of microbial classification levels was carried out, and PyNAST was used to perform multiple alignments of representative sequences. Vegan and Picante packages in the R software (version 2.15.3) were used to calculate the values of observed species, as well as the Shannon, Simpson, Chao1, ACE, Goods coverage, and PD indexes, and to draw the rarefaction curve (Oksanen et al., 2010; Webb, 2010). Combining the count of the same OTUs and the relative abundance of OTUs, the Bray–Curtis distance was calculated by Vegan software. Principal coordinates analysis (PCoA) was drawn by ape software. Tax4Fun was used to predict functional features based on 16S rRNA gene sequencing data (Aßhauer et al., 2015), and secondary metabolic pathways were clustered.

### Statistical Analyses

The data of this study were analyzed using one-way ANOVA of SPSS 25.0 (IBM, Armonk, NY, United States), in which the relative abundances of microbial phyla and genera were compared using Kruskal–Wallis test. The regression relationship between the relative abundance of bacteria and the level of dietary peNDF was analyzed, and Spearman correlation analysis between the relative abundance and the function of rumen bacteria was also executed. Tukey’s multiple test was used to compare differences among the treatment groups. Statistical significance was defined as *p* < 0.05, and trends were discussed at 0.05 < *p* < 0.10. Results are expressed as mean and standard error of the mean.

## Results

### Effects of Dietary Physically Effective Neutral Detergent Fiber Level on Feed Intake and Growth Performance

The effects of differing peNDF_1_._18_ contents in diets on the BW, DMI, and ADG of goats are presented in Table 2. DMI and ADG increased first and then decreased with decreasing peNDF1.18 content in the diets (*p* < 0.05), where the DMI of the 26.5% peNDF_1_._18_ treatment was significantly more than those of the 33.0, 29.9, and 24.8% peNDF_1_._18_ treatments (*p* < 0.05); the DMI of the 28.1% peNDF_1_._18_ treatment was not significantly different from those of other treatments. The ADG of the 26.5% peNDF_1_._18_ treatment was greater than that of 33.0%,and the ADG of the 24.8% treatment was significantly lower than those of other treatments.

**TABLE 2:** Effects of different contents of peNDF_1_._18_ in diets on the BW, ADG, and DMI of goats.

Items	Groups^1^					SEM	*P*-value
	H (33.0%)	MH (29.9%)	M (28.1%)	ML (26.5%)	L (24.8%)		
Initial BW (kg)	21.4	21.3	21.5	21.4	21.5	0.249	1.000
Final BW (kg)	24.6	24.9	25.2	26.0	24.1	0.332	0.480
DMI (g/day)	678^b^	704^b^	737^ab^	831^a^	702^b^	17.55	0.032
ADG (g/day)	70.6^b^	75.1^ab^	83.0^ab^	98.5^a^	58.7^c^	3.720	0.043

*^a–c^Means within a row with different superscripts differ significantly (p < 0.05).*

*^1^Groups: different peNDF_1_._18_ (PS > 1.18 mm) contents of 33.0, 29.9, 28.1, 26.5, and 24.8% were obtained by chopping or crusher crushing the forage into the following lengths: H (7 cm), MH (4 cm), M (1 cm), ML (5-mm sieve), and L (1-mm sieve).*

### Effects of Dietary Physically Effective Neutral Detergent Fiber Level on the Morphology of Goat Rumen

**FIGURE 1:**
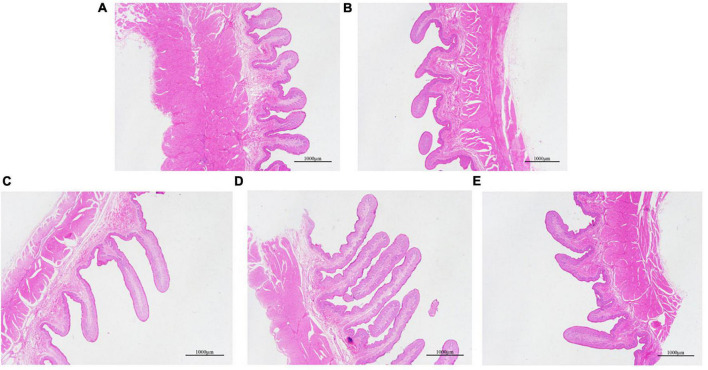
Representative image of the dorsal sac of rumen from five groups. **(A)** 33.0%, 7 cm, H group. **(B)** 29.9%, 4 cm, MH group. **(C)** 28.1%, 1 cm, M group. **(D)** 26.5%, 5 mm, ML group. **(E)** 24.8%, 1 mm, L group.

As shown in the results in Table 3, the level of dietary peNDF has a significant quadratic effect on the length of the rumen dorsal papilla and the width of the abdominal papilla, in which the values of 26.5 and 28.1% peNDF_1_._18_ treatments are the highest, respectively. The photographs of each are shown in Figure 1, where we choose five representative images of the dorsal sac of the rumen from each group.

**TABLE 3:** Effects of dietary peNDF level on the morphology of goat rumen.

Items	Groups^1^					SEM	*P*-value		
	H (33.0%)	MH (29.9%)	M (28.1%)	ML (26.5%)	L (24.8%)		ANOVA linear quadratic		
**Dorsal sac**									
Papilla length (mm)	1.015^c^	1.427^bc^	1.642^b^	2.281^a^	1.652^b^	0.205	< 0.001	< 0.001	0.003
Papilla width (mm)	0.560^ab^	0.487^b^	0.595^a^	0.533^ab^	0.588^a^	0.034	0.046	0.194	0.349
Muscle thickness (mm)	1.470^a^	1.327^b^	1.130^c^	1.446^ab^	1.210^c^	0.125	0.014	0.159	0.330
**Ventral sac**									
Papilla length (mm)	1.405	1.657	1.594	1.554	1.850	0.271	0.374	0.198	0.875
Papilla width (mm)	0.558^b^	0.583^ab^	0.664^a^	0.584^ab^	0.449^c^	0.031	< 0.001	0.003	< 0.001
Muscle thickness (mm)	1.237	1.155	1.423	1.199	1.493	0.131	0.069	0.061	0.452

*^a–c^Means within a row with different superscripts differ significantly (p < 0.05).*

*^1^Groups: different peNDF_1_._18_ (PS > 1.18 mm) contents of 33.0, 29.9, 28.1, 26.5, and 24.8% were obtained by chopping or crusher crushing the forage into the following lengths: H (7 cm), MH (4 cm), M (1 cm), ML (5-mm sieve), and L (1-mm sieve).*

### Data Acquired From Sequencing and Operational Taxonomic Unit Diversity

**FIGURE 2:**
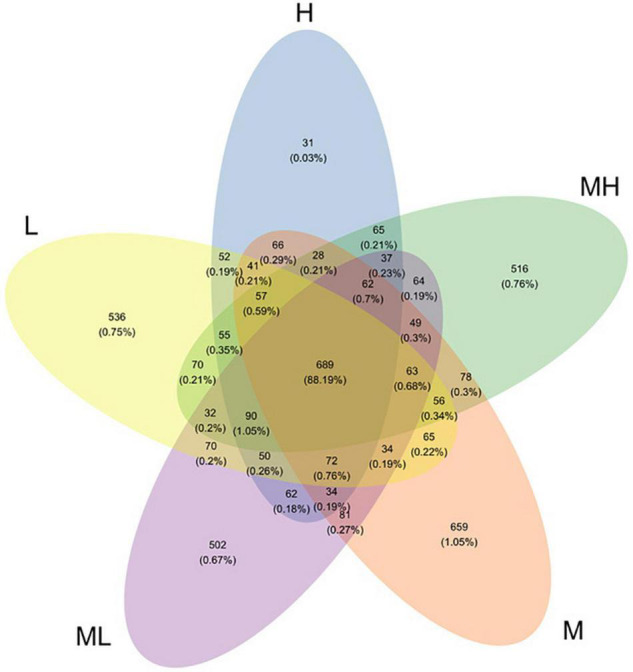
Venn diagram at the 97% similarity level. H group: 33.0% peNDF_1.18_ treatment. MH group: 29.9% peNDF_1.18_ treatment. M group: 28.1% peNDF_1.18_ treatment. ML group: 26.5% peNDF_1.18_ treatment. L group: 24.8% peNDF_1.18_ treatment.

The number of raw sequences in the rumen was 535,650 and the effective sequences were 509,933 based on the high-throughput sequencing analysis of 16S rRNA genes. The average effective ratio reached 90.36%. Clustering was based on the 97% sequence similarity from the effective sequences, where a total of 9,659 OTUs were obtained. The average OTUs in the 33.0, 29.9, 28.1, 26.5, and 24.8% peNDF_1_._18_ treatments were 1,491, 2,011, 2,134, 1,991, and 2,032, respectively. A total of 689 OTUs were shared across the five treatments, and the number of sequences in shared OTUs accounted for 88.19% of the total number of sequences (Figure 2).

The rarefaction curve (Supplementary Figure 1) was inclined to reach a plateau, and the value of coverage (Table 4) reached 0.96, both of which indicated that the sampling had sufficient sequence coverage to detect the majority of rumen bacteria.

**TABLE 4:** Effects of dietary peNDF_1_._18_ level on alpha diversity indexes of rumen bacteria in goats.

Items	Groups^1^					SEM	*P*-value
	H (33.0%)	MH (29.9%)	M (28.1%)	ML (26.5%)	L (24.8%)		
Chao1	1,179.535	1,473.747	1,485.104	1,544.153	1,535.871	189.289	0.344
ACE	1,203.733	1,524.217	1,538.947	1,593.639	1,584.490	204.956	0.350
Simpson	0.985^c^	0.990^ab^	0.993^a^	0.989^ab^	0.987^bc^	0.001	0.001
Shannon	5.391^b^	5.711^ab^	5.939^a^	5.672^ab^	5.702^ab^	0.144	0.043
PD	54.047	67.621	71.232	67.648	69.662	10.585	0.529
Coverage	0.972	0.963	0.965	0.960	0.961	0.006	0.392

*^a–c^Means within a row with different superscripts differ significantly (p < 0.05).*

*^1^Groups: different peNDF_1_._18_ (PS > 1.18 mm) contents of 33.0, 29.9, 28.1, 26.5, and 24.8% were obtained by chopping or crusher crushing the forage into the following lengths: H (7 cm), MH (4 cm), M (1 cm), ML (5-mm sieve), and L (1-mm sieve).*

### Alpha Diversity Analysis of Rumen Bacteria

Alpha diversity indexes include Shannon, Simpson, Chao1, ACE, and PD indexes. Chao1 and ACE indexes were similar among the treatments (Table 4), which meant that the total number of rumen bacteria did not differ. Shannon and Simpson indexes of the 28.1% peNDF_1_._18_ treatment were the highest (*p* < 0.05), indicating the highest richness and most even distribution of bacteria in the 28.1% peNDF_1_._18_ treatment. Dietary peNDF_1_._18_ levels had significant impacts on the diversity of goat rumen bacterial flora.

### Beta Diversity Analysis of Rumen Bacteria

**FIGURE 3:**
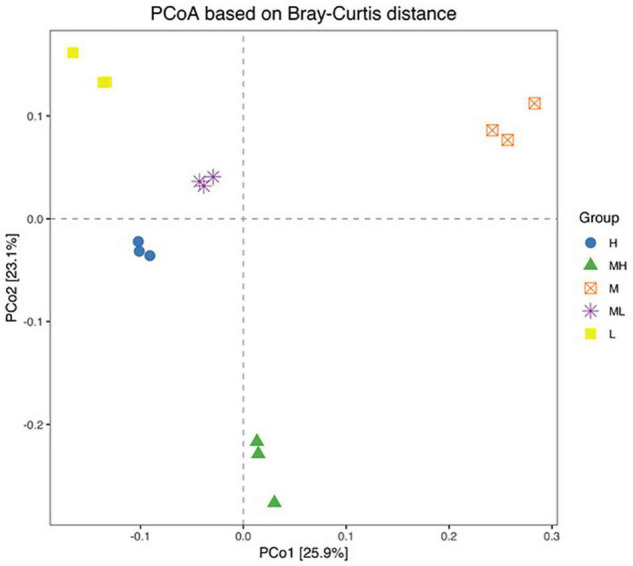
Bray–Curtis distance matrix PCoA of rumen bacterial samples. H group: 33.0% peNDF_1.18_ treatment. MH group: 29.9% peNDF_1.18_ treatment. M group: 28.1% peNDF_1.18_ treatment. ML group: 26.5% peNDF_1.18_ treatment. L group: 24.8% peNDF_1.18_ treatment.

A PCoA plot with the Bray–Curtis distance matrix was drawn to visualize the differences among the five groups (Figure 3). The percentage of variation was represented by PCo1 (25.9%) and PCo2 (23.1%); the closer the distance in the figure, the more similar the bacterial community composition of the sample. There was separation among groups, with the different structures of rumen bacteria shown. Permutational multivariate analysis of variance (PerMANOVA) was used to test the significance of differences between peNDF levels (Table 5), with significant differences (*p* < 0.001) between the five groups.

**TABLE 5:** PerMANOVA of rumen bacterial samples based on Bray–Curtis distances.

Items	Df	SumsOfSqs	MeanSqs	F.model	*R* ^2^	Pr (> F)
Group	4	0.901	0.225	8.440	0.771	< 0.001
Residuals	10	0.267	0.027	NA	0.228	NA
Total	14	1.168	NA	NA	1	NA

### Core Rumen Bacteria at Phylum and Genus Levels

**FIGURE 4:**
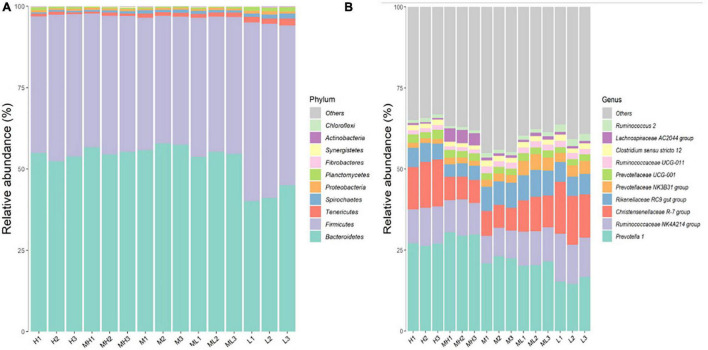
Rumen bacterial compositions **(A)** at the phylum level (only the top 10 abundant phyla were presented) and **(B)** at the genus level (only the top 10 abundant genera were presented). H group: 33.0% peNDF_1.18_ treatment. MH group: 29.9% peNDF_1.18_ treatment. M group: 28.1% peNDF_1.18_ treatment. ML group: 26.5% peNDF_1.18_ treatment. L group: 24.8% peNDF_1.18_ treatment.

In this experiment, 22 phyla and 242 genera were taxonomically classified. The dominant phyla of goat rumen bacteria were Bacteroidetes (42.07–57.01%) and Firmicutes (39.77–52.51%) (Figure 4). The dominant phylum transitioned from Bacteroidetes to Firmicutes with the decrease of dietary peNDF_1_._18_ level. In other words, the relative abundance of Bacteroidetes decreased with the decrease of dietary peNDF_1_._18_ level, while the trend of changes in Firmicutes was the opposite. The relative abundances of Tenericutes, Spirochetes, and Planctomycetes were the highest in the 24.8% peNDF_1_._18_ treatment (*p* < 0.05).

The top three dominant genera of goat rumen bacteria were *Prevotella 1* (average, 23.00%), *Ruminococcaceae NK4A214 group* (average, 10.70%), and *Christensenellaceae R-7 group* (average, 10.61%). The relative abundance of *Prevotella 1* was the highest in the 29.9% peNDF_1_._18_ treatment and the lowest in the 24.8% peNDF_1_._18_ treatment (*p* < 0.001). The relative abundances of *Ruminococcaceae NK4A214 group* and *Christensenellaceae R-7 group* decreased when peNDF level increased from 24.8 to 28.1%, and then increased with dietary peNDF level. An uncultured genus, f_F082| g_*uncultured*, subordinated to Bacteroidetes, was also one of the predominant genera.

**FIGURE 5:**
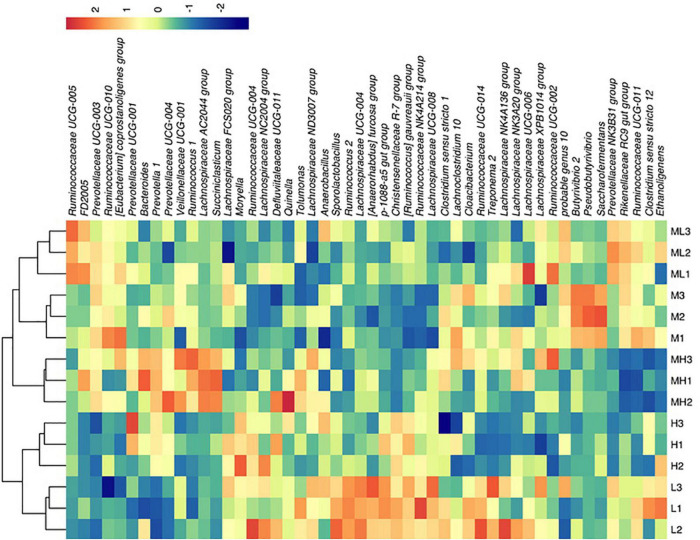
Heat map of species abundance at the genus level (top 50 bacteria). H group: 33.0% peNDF_1.18_ treatment. MH group: 29.9% peNDF_1.18_ treatment. M group: 28.1% peNDF_1.18_ treatment. ML group: 26.5% peNDF_1.18_ treatment. L group: 24.8% peNDF_1.18_ treatment. The abscissa is sorted according to the order of the samples, and the ordinate is arranged according to the total average abundance from top to bottom. The relative abundance of the genus is not converted, and the legend shows the percentage. The redder color represents higher abundance, and the bluer color represents lower abundance.

According to the species annotation and relative abundance information of all samples at the genus level, the top 50 abundant genera were selected for clustering and plotted into a heat map (Figure 5). The 28.1 and 26.5 peNDF_1_._18_ treatments were grouped into one cluster first and then grouped into one cluster with the 29.9% peNDF_1_._18_ treatment, which meant that the composition of these three groups was different from the 24.8 to 33.0% peNDF_1_._18_ treatments.

### Comparisons of Rumen Bacterial Composition Among the Five Groups

Bacteria with a phylum level greater than 1% and a genus level more than 0.1% were selected for differential analysis (Zened et al., 2013). We found that the bacterial flora of the rumen solid contents was significantly affected by dietary peNDF_1_._18_ levels (Table 6). At the phylum level, the relative abundances of Bacteroidetes, Firmicutes, Tenericutes, Spirochetes, and Planctomycetes were affected by dietary peNDF_1_._18_ levels; besides, the relative abundance of Proteobacteria (average, 0.654%) was also different (*p* = 0.04) and that in the 24.8% peNDF_1_._18_ treatment was the highest. At the genus level, in addition to the three dominant genera, there were also another 20 genera that significantly differed among groups, and most of them belong to Firmicutes. The relative abundance of many uncultured bacteria genera (belonging to Bacteroidetes) also differed greatly among treatments.

**TABLE 6:** Effects of dietary peNDF_1_._18_ level on the relative abundance of rumen bacteria in goats.

Items	Groups^1^					SEM	*P*-value
	H (33.0%)	MH (29.9%)	M (28.1%)	ML (26.5%)	L (24.8%)		
**Phylum (%)**							
Bacteroidetes	53.713^a^	55.471^a^	57.010^a^	54.551^a^	42.073^b^	1.224	< 0.001
Firmicutes	43.572^b^	41.801^b^	39.772^b^	42.100^b^	52.510^a^	1.321	0.019
Tenericutes	0.779^b^	0.789^b^	1.173^b^	1.275^b^	1.821^a^	0.154	< 0.001
Spirochetes	0.470^b^	0.621^b^	0.887^ab^	0.763^b^	1.259^a^	0.136	0.002
Planctomycetes	0.680^b^	0.355^b^	0.414^b^	0.421^b^	1.157^a^	0.117	0.033
**Genus (%)**							
*Prevotella 1*	26.776^b^	29.889^a^	22.167^c^	20.669^c^	15.518^d^	0.696	< 0.001
*Rikenellaceae RC9 gut group*	5.571^b^	4.105^c^	7.484^a^	7.905^a^	6.117^b^	0.276	< 0.001
*Prevotellaceae NK3B31 group*	1.482^c^	1.795^c^	2.347^c^	4.510^a^	3.468^b^	0.291	< 0.001
*Prevotellaceae UCG-001*	2.449^a^	2.225^a^	1.992^b^	2.146^ab^	1.910^b^	0.206	0.037
*Prevotellaceae UCG-003*	0.881^b^	1.479^a^	1.505^a^	1.426^a^	0.937^b^	0.084	< 0.001
*Ruminococcus 1*	0.651^b^	1.308^a^	0.920^b^	0.917^b^	0.631^b^	0.106	0.001
*Ruminococcus 2*	1.091^bc^	0.812^c^	1.131^bc^	1.236^b^	2.209^a^	0.108	0.003
*Ruminococcaceae UCG-002*	0.230^b^	0.427^a^	0.345^ab^	0.437^a^	0.342^ab^	0.086	0.022
*Ruminococcaceae UCG-004*	0.289^ab^	0.197^ab^	0.141^b^	0.194^ab^	0.306^a^	0.046	0.022
*Ruminococcaceae UCG-005*	0.513^b^	0.457^b^	0.529^b^	1.440^a^	0.306^b^	0.070	< 0.001
*Ruminococcaceae UCG-010*	0.569^ab^	0.638^ab^	0.818^a^	0.746^ab^	0.450^b^	0.099	0.029
*Ruminococcaceae UCG-011*	1.335^b^	1.167^b^	1.897^a^	1.831^a^	1.828^a^	0.113	< 0.001
*Ruminococcaceae UCG-014*	0.559^c^	1.052^b^	0.894^bc^	0.703^bc^	1.538^a^	0.116	< 0.001
*Ruminococcaceae NK4A214 group*	11.212^ab^	10.255^bc^	8.576^c^	10.476^bc^	12.957^a^	0.674	0.001
*Lachnospiraceae AC2044 group*	0.519^c^	3.990^a^	0.861^bc^	0.914^b^	0.786^bc^	0.108	< 0.001
*Lachnospiraceae UCG-004*	0.424^bc^	0.486^b^	0.293^c^	0.299^c^	1.206^a^	0.055	< 0.001
*Lachnospiraceae UCG-008*	0.615^b^	0.631^b^	0.319^c^	0.440^bc^	0.927^a^	0.124	< 0.001
*Christensenellaceae R-7 group*	13.871^a^	7.129^c^	7.240^c^	10.002^b^	14.791^a^	0.608	< 0.001
*Butyrivibrio 2*	0.789^b^	1.009^b^	1.969^a^	1.049^b^	0.976^b^	0.144	0.049
*Moryella*	0.595^a^	0.273^b^	0.309^b^	0.273^b^	0.437^ab^	0.072	0.005
*Pseudobutyrivibrio*	0.171^b^	0.247^b^	1.788^a^	0.342^b^	0.174^b^	0.103	< 0.001
*Sporolactobacillus*	1.022^ab^	0.874^b^	0.878^b^	0.904^b^	1.512^a^	0.156	0.010
*Treponema 2*	0.595^a^	0.273^b^	0.309^b^	0.273^b^	0.437^ab^	0.072	0.005
*p-1088-a5 gut group*	0.289^ab^	0.197^ab^	0.141^c^	0.194^ab^	0.306^a^	0.046	0.036
**Uncultured (%)**							
f_F082| g_*uncultured*	10.111^a^	6.853^c^	10.397^a^	8.507^b^	7.774^bc^	0.284	< 0.001
f_Muribaculaceae| g_*uncultured*	3.389^b^	5.085^a^	6.045^a^	5.877^a^	3.100^b^	0.292	0.015
f_Bacteroidales RF16 group| g_*uncultured*	0.483^bc^	0.772^a^	0.667^ab^	0.546^b^	0.306^c^	0.057	< 0.001
f_Bacteroidales BS11 gut group| g_*uncultured*	0.243^b^	0.782^a^	0.713^a^	0.332^b^	0.358^b^	0.065	< 0.001
f_Paludibacteraceae| g_*uncultured*	0.365^b^	0.427^ab^	0.546^a^	0.460^ab^	0.398^ab^	0.046	0.034
f_p-251-o5| g_*uncultured*	0.463^ab^	0.197^c^	0.634^a^	0.289^bc^	0.424^b^	0.055	< 0.001
f_Lachnospiraceae| g_*uncultured*	0.930^b^	1.134^ab^	1.292^a^	0.832^b^	1.134^ab^	0.093	0.004
o_Mollicutes RF39| f_uncultured	0.772^b^	0.782^b^	1.150^b^	1.256^b^	1.814^a^	0.153	< 0.001

*^a–c^Means within a row with different superscripts differ significantly (p < 0.05).*

*^1^Groups: different peNDF_1_._18_ (PS > 1.18 mm) contents of 33.0, 29.9, 28.1, 26.5, and 24.8% were obtained by chopping or crusher crushing the forage into the following lengths: H (7 cm), MH (4 cm), M (1 cm), ML (5–0 mm sieve), and L (1-mm sieve).*

### Regression Analysis Between peNDF_1_._18_ Level and Relative Abundance of Rumen Bacteria

The relationships between dietary peNDF_1_._18_ levels (x) and the relative abundance (y) of rumen bacteria at the phylum or genus level are summarized in Table 7. We found that there was a significant quadratic correlation (*p* < 0.001) between most of the bacterial flora and dietary peNDF_1_._18_ levels, and that the level of dietary peNDF_1_._18_ had the greatest impact on the relative abundance of Tenericutes and *Christensenellaceae R-7 group*, with high fitness (*R*^2^ was 0.804 and 0.958, respectively).

**TABLE 7:** Regression relationship between dietary peNDF_1_._18_ level (x) and relative abundance of rumen bacteria (y) (*n* = 15, *R*^2^ > 0.6).

Items	Regression equation	*R* ^2^	*P*-value
**Phylum**			
Bacteroidetes	y = –55.129x^2^ + 32.985x–4.327	0.795	< 0.001
Firmicutes	y = 47.844x^2^–28.484x + 4.657	0.745	< 0.001
Tenericutes	y = 2.009x^2^–1.287x + 0.215	0.804	< 0.001
Spirochetes	y = 0.848x^2^–0.576x + 0.104	0.622	0.001
Planctomycetes	y = 3.534x^2^–2.087x + 0.313	0.729	< 0.001
**Genus**			
*Prevotella 1*	y = –29.128x^2^ + 18.351x–2.590	0.851	< 0.001
*Ruminococcaceae NK4A214 group*	y = 16.860x^2^–9.899x + 1.555	0.604	0.002
*Christensenellaceae R-7 group*	y = 44.347x^2^–25.763x + 3.818	0.958	< 0.001
*Ruminococcus 2*	y = 4.394x^2^–2.665x + 0.414	0.885	< 0.001
*Treponema 2*	y = 0.486x^2^–0.357x + 0.071	0.638	0.001
*Moryella*	y = 1.452x^2^–0.820x + 0.119	0.660	0.001
*p-1088-a5 gut group*	y = 3.644x^2^–2.140x + 0.318	0.765	< 0.001
*Prevotellaceae UCG-003*	y = –3.735x^2^ + 2.147x–0.292	0.884	< 0.001
**Uncultured**			
o_Mollicutes RF39| f_uncultured	y = 2.068x^2^–1.319x–0.220	0.807	< 0.001
f_Muribaculaceae| g_*uncultured*	y = –15.459x^2^ + 8.895x–1.226	0.763	< 0.001
f_Bacteroidales RF16 group| g_*uncultured*	y = –2.003x^2^ + 1.182x–0.166	0.846	< 0.001

### Prediction of Rumen Microbial Flora Function

According to the prediction of microbial function by the Tax4Fun program and the SILVA database, the gene functions of each group in annotation level 2 were selected to perform differential analysis. We found that dietary peNDF_1_._18_ levels had significant effects on the prediction of rumen bacterial function (Table 8). Table 8 shows that the main gene functions of rumen bacteria are associated with carbohydrate metabolism, amino acid metabolism, and membrane transport.

**TABLE 8:** Effects of dietary peNDF_1_._18_ level on the prediction of rumen bacterial function (%).

Functions	Groups^1^					SEM	*P*-value
	H (33.0%)	MH (29.9%)	M (28.1%)	ML (26.5%)	L (24.8%)		
**Metabolism**							
Nucleotide metabolism	7.190^a^	7.103^b^	7.064^b^	7.112^ab^	7.095^b^	0.025	0.006
Amino acid metabolism	10.654^d^	10.693^cd^	10.962^b^	10.836^bc^	11.196^a^	0.044	< 0.001
Metabolism of other amino acids	2.146^ab^	2.154^a^	2.138^b^	2.151^ab^	2.110^c^	0.004	< 0.001
Carbohydrate metabolism	15.868^ab^	15.778^b^	15.811^b^	16.026^a^	15.511^c^	0.062	< 0.001
Glycan biosynthesis and metabolism	4.415^a^	4.228^bc^	4.196^c^	4.397^ab^	3.979^d^	0.056	< 0.001
Lipid metabolism	2.810^b^	2.815^b^	2.822^ab^	2.853^a^	2.752^c^	0.011	< 0.001
Energy metabolism	6.636^c^	6.648^bc^	6.681^ab^	6.681^abc^	6.706^a^	0.014	0.003
**Genetic information processing**							
Translation	6.651^a^	6.526^b^	6.425^bc^	6.497^b^	6.320^c^	0.033	< 0.001
Replication and repair	6.310^a^	6.202^b^	6.078^c^	6.170^bc^	5.951^d^	0.032	< 0.001
Folding, sorting, and degradation	2.947^a^	2.902^ab^	2.879^b^	2.914^ab^	2.828^c^	0.015	< 0.001
**Environmental information processing**							
Membrane transport	8.197^c^	8.644^b^	8.455^bc^	8.056^c^	9.085^a^	0.125	< 0.001
Signal transduction	6.024^c^	6.165^bc^	6.346^ab^	6.170^bc^	6.479^a^	0.067	< 0.001
**Cellular processes**							
Cell motility	1.724^bc^	1.881^a^	1.828^ab^	1.669^c^	1.962^a^	0.046	0.001
Cell growth and death	1.808^a^	1.799^ab^	1.791^b^	1.790^b^	1.796^b^	0.003	0.002
**Organismal systems**							
Endocrine system	0.422^a^	0.415^ab^	0.404^b^	0.420^a^	0.378^c^	0.004	< 0.001
Immune system	0.143^ab^	0.133^c^	0.135^bc^	0.144^a^	0.123^d^	0.003	< 0.001
Digestive system	0.534^a^	0.484^c^	0.487^bc^	0.530^ab^	0.427^d^	0.013	< 0.001
**Human diseases**							
Infectious diseases	1.645^a^	1.641^a^	1.616^b^	1.617^b^	1.564^c^	0.004	0.043

*^a–d^Means within a row with different superscripts differ significantly (p < 0.05).*

*^1^Groups: different peNDF_1_._18_ (PS > 1.18 mm) contents of 33.0, 29.9, 28.1, 26.5, and 24.8% were obtained by chopping or crusher crushing the forage into the following lengths: H (7 cm), MH (4 cm), M (1 cm), ML (5-mm sieve), and L (1-mm sieve).*

## Discussion

### Growth Performance and Rumen Development

Compared with pigs and poultry, goats, as ruminants, can better use fiber in feed as energy source for their growth and development. Therefore, evaluation of dietary fiber, especially NDF (peNDF), is crucial for the nutritional value of goats or other ruminants. In this study, hay was ground into different lengths to obtain five peNDF_1_._18_ levels in diets, which have an impact on goat DMI. When peNDF_1_._18_ > 26.5%, as peNDF declined, goat DMI increased, which is consistent with the research of Park et al. (2014). Wang et al. (2017) observed that body growth improved with increasing peNDF_8_._0_. Although heifers’ DMI did not change significantly among different peNDF_8_._0_ contents in the study of Wang et al. (2017) and in another study concluded by Beauchemin and Yang (2005), in the present work and in this study, the DMI and ADG of the 26.5% peNDF_1_._18_ treatment were significantly greater than those of 33.0 and 24.8% peNDF_1_._18_ treatments (Jang et al., 2017). The different results of the study may be attributed to the differences in the animals and in the diets: goats are smaller than cows, so their sensitivity to peNDF may be higher than that of cattle. In a research conducted by Yang et al. (2017), they found that the differences in the ADG of goats among the treatment groups are generally higher than those in this study, which is also due to the smaller body size of goats in the study of Jang et al. (2017). Particles with a length of 10 mm were retained in the reticulo-rumen 19–28 h longer than 1-mm-long particles of the same density. Multiple regression analysis indicated that particle density and PS accounted for 59 and 28% of the total variation of mean retention time in the reticulo-rumen, respectively (Ehle et al., 1984; Kaske and Engelhardt, 1990).

The level of dietary fiber is extremely important for the growth and development of the gastrointestinal tract of young ruminants, while the length and width of the rumen papilla are especially vital indicators of the degree of rumen development (Lesmeister et al., 2004). The experimental results of Beiranvand et al. (2014) showed that 2.6-mm alfalfa hay could increase the thickness of the rumen muscle of calves and reduce the width of the nipple. Xu et al. (2010) found that feeding whole corn reduced the height and width of the rumen papilla of goats compared to when feeding goat with fine, coarse, and whole corn. The results of this study show that the 28.1 and 26.5% peNDF_1_._18_ treatments are more beneficial to the development of rumen papilla. In other words, moderate peNDF level is conducive to the development of the gastrointestinal tract of goats.

### Rumen Bacterial Community

In this study, we attempted to study the impact of dietary peNDF_1_._18_ level on the rumen bacterial flora of goat and discovered that rumen bacteria could be affected by just changing the PS of forage. Results in Shannon and Simpson indexes indicated that the level of dietary peNDF_1_._18_ was a strong determinant of rumen microbial community composition in goats. Previous studies reported that the high diversity of microorganisms was beneficial for the production performance of animals (Arrazuria et al., 2016; Yuan et al., 2019). Similarly, high microbial diversity is linked to good meat and wool production in sheep (Zeng et al., 2017), which is the common ruminant. In this experiment, the microbial diversity indexes between peNDF_1_._18_ treatments implied that the peNDF_1_._18_ levels between 26.5 and 29.9% were perhaps more beneficial to goat health. Zebeli et al. (2008) and Valizadeh et al. (2010) also found that dietary peNDF level did not affect the number of rumen bacteria by absolute quantification of rumen bacteria. Moreover, the level of dietary peNDF_1_._18_ was a strong determinant of rumen microbial community structure, according to the results of PCoA profiling and PerMANOVA. Previous studies on cow and dairy goat also showed that the PS of the feedstuff influenced the relative abundance of rumen (Tajima et al., 2001; Li et al., 2018a).

Previous studies reported that the dominant phyla in the rumen were usually Bacteroidetes, Firmicutes, and Proteobacteria (Shabat et al., 2016). The fiber in diets has recently been proved to be a factor affecting microbiota in the gastrointestinal tract. Liu et al. (2017) discovered that the relative abundance of Proteobacteria in goat rumen would increase with the decrease of feed fiber content. Sika deer fed oak-leave-based diets had a higher relative abundance of Bacteroidetes than deer fed corn-stalk-based diets (Li et al., 2018b). Crowley et al. (2017) found that the relative abundance of Bacteroidetes increased when rabbits were fed a diet with a higher fiber content. However, there were only limited studies on the effects of roughage length on rumen microbiota. One study conducted by Li et al. (2018a) found that both roughage and concentration length influence the rumen microbiota. Specifically, this study found that as the level of dietary peNDF_1_._18_ decreased, the dominant phylum transitioned from Bacteroidetes to Firmicutes. The shorter the forage, the higher was the relative abundance of Proteobacteria, which was consistent with the results in rabbits (Yuan et al., 2019). Existing studies have shown that increased relative abundance of Proteobacteria is a common feature of health impairment (Perea et al., 2017), suggesting that the peNDF_1_._18_ level of goat diet should not be less than 24.8%.

Shen et al. (2017) reported that the relative abundance of Tenericutes in goat rumen increased with the level of concentrate. Yuan et al. (2019) found that the relative abundance of Tenericutes in the caecum of rabbits was negatively correlated to the PS of alfalfa (Yuan et al., 2019), which was similar to our study. Spirochetes, a kind of fiber-degrading phylum, was negatively affected by the dietary addition of starch or oil (Zened et al., 2013). Our results proved that the relative abundance of Spirochetes was highest in the 24.8% peNDF_1_._18_ treatments. More studies are needed to explore the reasons for the changes in the relative abundance of phyla with dietary peNDF and to explore the functions of these predominant phyla.

As for the dominant genera of rumen bacteria, a large number of reports point out that *Prevotella* is the dominant genus in the rumen (Ramírez-Restrepo et al., 2016) and that its main function is protein degradation (Myer et al., 2015). Moreover, Satoshi et al. (2010) found that *Prevotella* was also involved in fiber degradation. Bekele et al. (2010) found that *Prevotella* accounted for 56 or 60% of goat rumen bacteria and that its relative abundance was even higher when goats were fed hay diet than when fed concentrate diet. Huo et al. (2014) found that *Prevotella* accounted for less than 20% of goat rumen bacteria, which was close to our results. Huo et al. (2014) also discovered that a high-grain diet was not good for the growth of *Prevotella*. Our study, for the first time, disclosed that the growth of *Prevotella* was sensitively affected by forage length; fine-crushed forage (1 mm) was not conducive to the growth of *Prevotella 1* and *Prevotellaceae UCG-001*; and the optimal level of peNDF_1_._18_ for the growth of *Prevotellaceae NK3B31 group* and *Prevotellaceae UCG-003* was 26.5% as judged by the relative abundance. Thus, we could conclude that dietary peNDF affects the growth of *Prevotella* at different patterns according to the different Prevotellaceae isolates. Spearman correlation analysis showed that the relative abundance of *Prevotellaceae UCG-001* was negatively related, but that of *Prevotellaceae NK3B31 group* was positively related, to amino acid metabolism. This implied that rumen bacteria at the genus level may be too rough to study the function of the bacteria, and isolates under the genus would be accurate enough to investigate the functions. A previous study has shown that *Prevotella* is related to protein degradation (Myer et al., 2015); however, we found that the relative abundance of *Prevotella 1* (the dominant *Prevotella* in goat rumen) was negatively related to amino acid metabolism, and that the apparent digestibility of crude protein and the relative abundance of *Prevotella 1* were similarly the lowest in the 24.8% peNDF_1_._18_ treatments. This result shows that the function of a single strain cannot represent the role of bacteria at the genus level. Moreover, there is still a gap between the predicted functions and the actual situation, and more technical methods are needed to study the specific functions of bacteria.

*Ruminococcus* is a major cellulolytic genus (Bryant, 1959). In our study, the relative abundance of *Ruminococcaceae* isolates did not change orderly with dietary peNDF_1_._18_ levels, but the relative abundances of *Ruminococcaceae NK4A214 group* and *Ruminococcus 2* were quadratically related to the level of dietary peNDF_1_._18_ (Table 6). Pitta et al. (2014) found that increasing the proportion of fiber in buffalo diet could increase the relative abundance of *Ruminococcus*. Zebeli et al. (2008) found that the count of *Ruminococcus* was not affected by dietary PS *via* real-time quantitative PCR. However, our experiment showed that the count of *Ruminococcus* was affected by peNDF by high-throughput sequencing, and different *Ruminococcus* isolates (*Ruminococcus 1* and *2*) responded differently to peNDF levels (Table 5), reminding us to be species-specific when studying dietary effects on bacteria. *Christensenella* was also a kind of dominant genus in goat rumen, and its relative abundance varied with the different treatments, but Spearman correlation analysis showed that it was not significantly related to the top 10 functions of bacteria (*p* > 0.05). In other words, their functions might be minor for host metabolism. Zhang et al. (2017) also concluded that *Christensenella* possessed little function in the rumen of Holstein heifer, but they attributed this to the low relative abundances.

*Butyrivibrio*, *Clostridium*, and *Fibrobacter* are the main fiber-degrading bacteria in the rumen (Klieve et al., 2007; Zhang et al., 2017), and many members of *Lachnospiraceae* also have cellulolytic activities (Nyonyo et al., 2014). Zhang et al. (2017) found that the relative abundance of *Fibrobacter* (0.36–2.35%) in the rumen of Holstein heifer elevated with the increase in the concentrate level of their diet. Our result showed that the relative abundance of *Fibrobacter* in goat rumen was less than 0.1% and reached the peak when peNDF_1_._18_ was 24.8% (24.8% peNDF_1_._18_ treatment). This difference might be caused by the differences in animal species and diet (Metzler-Zebeli et al., 2013; Jin et al., 2016), but it could also be caused by differences in sequencing technology and primers. In this study, the relative abundance of *Clostridium* in the rumen of goat ranged from 1.27 to 1.97%, very similar to that in the rumen of sheep (Nyonyo et al., 2014), and was not affected by the level of peNDF_1_._18_. We found that the function of *Clostridium sensu stricto 12* was most likely in the metabolism of cofactors and vitamins instead of carbohydrates (Figure 5). Li et al. (2019) found that *Clostridium* could be involved in fiber fermentation. The different functions between the two reports were possibly due to the specific strain of *Clostridium*, but the deeper reason remains to be studied. The relative abundance of *Butyrivibrio 2* reached the summit at 28.1% peNDF_1_._18_ treatment (*p* = 0.049), which signified that dietary peNDF_1_._18_ deviating from 28.1% might reduce fiber digestion. Previous studies have shown that *Butyrivibrio 2* was negatively related to immune factors (IL-6 and IL-1β) (Li et al., 2019) and was also a considerable butyrate producer (Nyonyo et al., 2014) and a regulator of hemicellulose-degrading enzyme secretion (Dunne et al., 2012). Thus, it is necessary to study the relationship between relative abundance and host health, as well as production performance.

According to the correlation analysis between the relative abundance of bacteria, changing trends in Bacteroidetes and Firmicutes, and that of the genera under the two phyla, were inverse. In addition, the regression relationship between peNDF_1_._18_ level and the relative abundance of *Ruminococcaceae NK4A214 group* and *Prevotella 1* was inverse. Functional prediction explored that *Prevotella 1* and *Ruminococcaceae UCG-011* were in opposing camps, suggesting that the two bacteria may alter host nutrition in the opposite direction, but further studies are needed to explore the optimal relative abundance of the two genera and their functions in nutrient metabolism in goat rumen. Moreover, three uncultured bacteria were quadratically related to dietary peNDF_1_._18_ level, especially f_Muribaculaceae| g_*uncultured*, but this genus showed no correlation with the predicted function, so the specific function remains confirmatory. Overall, although a quadratic correlation exists, follow-up research experiments should be carried out on the premise of animal health and welfare. One of the shortcomings of this study was the small number of repetitions, which is worth noting in future experiments.

Existing studies have shown that gastrointestinal microbes have a strong correlation with the metabolism of the host (Nieuwdorp et al., 2014; Shi et al., 2017). In this study, we detected the main functions of rumen bacteria, and most genes were closely related to carbohydrate metabolism. The lowest function percentage in the 24.8% peNDF_1_._18_ treatment suggested that low dietary peNDF may not be beneficial to carbohydrate metabolism activities of rumen microorganisms. In terms of the metabolism of carbohydrates, amino acids, and energy, the range of dietary peNDF_1_._18_ between 26.5 and 28.1% seemed more beneficial to the metabolic activity of the host. We also found that the relative abundances of *Prevotella 1*, *Prevotellaceae UCG-001*, and *Ruminococcaceae UCG-011* were significantly related to the majority of the dominant functions, so it is important to study their functions in the future.

## Conclusion

This study proved that the differences in growth performance and rumen development were associated with changes in the rumen bacterial community. Based on the results of growth performance, rumen development, and rumen bacterial community, a peNDF level of between 26.5 and 28.1% in the diet was optimal for goats.

## Data Availability Statement

The datasets generated for this study can be found in online repositories. The names of the repository/repositories and accession number(s) can be found at: https://www.ncbi.nlm.nih. gov/, PRJNA693130.

## Ethics Statement

The animal study was reviewed and approved by the Animal Policy and Welfare Committee of Animal Nutrition Institute, Sichuan Agriculture University.

## Author Contributions

BCX and MW performed the experiments, analyzed the statistical data, prepared the figures and tables, reviewed earlier versions of the manuscripts, and approved the final manuscript. AH and XL carried out the experiments and gave tremendous help in data analysis. BX and QH designed and supervised the experiments. BX and SY were also in charge of preparing the manuscript. ZW, LW, and QP gave guidance on statistical analysis and on the plan of this study. All authors read and approved the final manuscript.

## Conflict of Interest

The authors declare that the research was conducted in the absence of any commercial or financial relationships that could be construed as a potential conflict of interest.

## Publisher’s Note

All claims expressed in this article are solely those of the authors and do not necessarily represent those of their affiliated organizations, or those of the publisher, the editors and the reviewers. Any product that may be evaluated in this article, or claim that may be made by its manufacturer, is not guaranteed or endorsed by the publisher.

## Footnotes

1
http://www.drive5.com/uparse/

2
http://www.arb-silva.de/
