# Supplementary material for: Changes in Rumen Bacterial Community Induced by the Dietary Physically Effective Neutral Detergent Fiber Levels in Goat Diets
Source: Front Microbiol. 2022 Apr 11;13:820509. doi: 10.3389/fmicb.2022.820509 (PMC9035740; doi:10.3389/fmicb.2022.820509)
Supplement: Supplementary file 1 [file Data_Sheet_1.docx]

**Table S1.** Ingredients and nutritional level of the diets (DM basis).

| Item | Content |
| --- | --- |
| Diet ingredient, % of DM |  |
| Ground corn | 37.10% |
| Soybean meal | 2.30% |
| Wheat bran | 2.90% |
| Alfalfa hay | 10.00% |
| Peanut vine | 9.00% |
| Leymus chinensis | 36.00% |
| CaCO_3_ | 0.17% |
| CaHPO_4`_2H_2_O | 1.02% |
| NaCl | 0.51% |
| Premix^1^ | 1.00% |
| Nutritional level^2^, % of DM |  |
| ME (MJ/kg) | 10.23 |
| CP | 8.87% |
| Ca | 0.74% |
| P | 0.87% |
| NDF | 42.89% |
| ADF | 30.50% |

^1^Premix (per kg) contains: Cu 1800 mg, Fe 4000 mg, Zn 6500 mg, Mn 8000 mg, I 100 mg, Se 5 mg, Vitamin A 800,000 IU, Vitamin D 120,000 IU, Vitamin E 50,000 IU, Vitamin K_3_ 200 mg, Vitamin B_1_ 200 mg, Vitamin B_12_ 5 mg.

^2^ME is calculated, the remaining indicators are measured values.


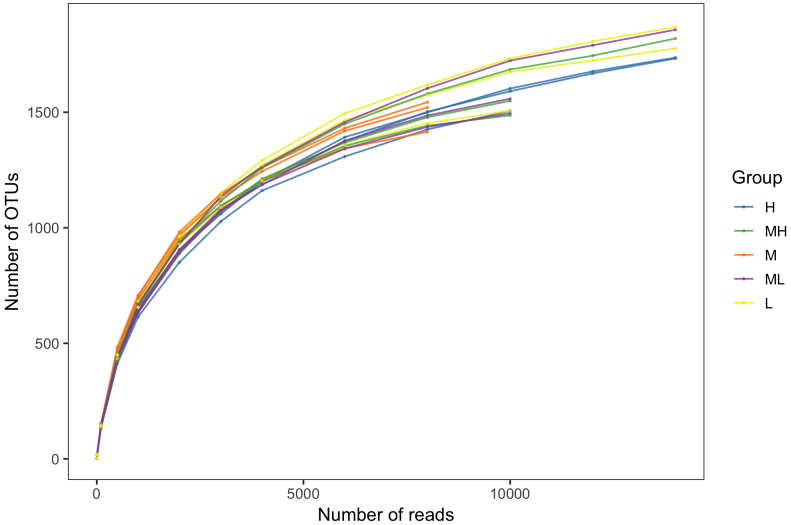


Figure S1. Rarefaction curves of each sample in different groups. (A): 33.0% 7cm H group. (B): 29.9% 4cm, MH group. (C): 28.1% 1cm M group. (D): 26.5% 5mm ML group and (E): 24.8% 1mm, L group.
